# Selections and Abstracts

**Published:** 1917-01

**Authors:** 


### SELECTIONS AND ABSTRACTS

### A FORM OF FOOD WASTE WHICH SHOULD BE CURTAILED.

The following statement by Lord Devonport deserves the most careful study, especially in these times of high food prices:

"The British government has issued an order reducing the quantity of beer to be brewed from April 1st to 70 per cent. of the output of the preceding year. This means a reduction to one-half of that of the year preceding the war. There is to be a corresponding restriction in the release of wine spirits in bond.

"The food controller, Lord Devonport, states that these steps are in no way to be deemed measures of temperance or social reform. The barley, sugar and other ingredients used in brewing are required for food. It is a question of bread versus beer. In the year preceding the war, ending March 31, 1914, the quantity of beer brewed amounted to 36,000,000 standard barrels. The production of beer will be restricted by the new order to 18,200,000 standard barrels. The food side of the reduction is represented by 286,000 tons of barley, 36,000 tons of sugar and 16,500 tons of grits, also a brewing ingredient. On the transport side there will be first of all, a large saving in mercantile marine tonnage; and, further, a considerable saving in land transport, such as railway and other forms of haulage. There will also be a consequent reduction in the amount of labor employed and fuel consumed in the process of manufacturing beer. This restriction of brewing will further have a direct favorable effect on the production of meat and milk, because it will set free for the use of agriculturists—the class which produces meat and milk—a greater percentage of offals (the residuum of the barley used in brewing) than at present produced from brewers' grains. At present the brewer returns 25 per cent. of the barley he uses in the shape of feeding offals. But the barley, when milled—as it is intended to be by this order—gives, in the first place, 50 per cent. of flour, which is, of course, direct human food, and, of the remainder, 40 per cent. is returned in the shape of what is termed millers' offals. The liquor interest has asserted both by advertisement and by misinformed press statement that any restriction of brewing would have the injurious direct effect on feeding stuffs used by agriculturists. As a matter of fact, under these restrictions, the contrary will be the case.''—Journ. Amer. Med. Assn.

According to figures compiled by the United States Internal Revenue Office, the production of beer in the City of New York reaches the enormous total of approximately 8,000,000 barrels annually. This is based on the standard barrel containing 31 gallons. Judging by the calculations made by the British experts this represents 127,000 tons of barley, 16,000 tons of sugar, and 7,300 tons of grits—nearly all wasted!

Does anyone still seriously deny that the time has come when this enormous waste shall be curtailed?

''More bread and less beer?'' This is the cry of all reasonable people.—Department of Health, City of New York.

---

### BIRTH AND DEATH RATES IN 1915.

Washington, D. C., January 17, 1917.—In the recently established birth-registration area of the United States—comprising the six New England states, New York, Pennsylvania, Michigan, Minnesota, and the District of Columbia, with an estimated population of 31,150,000, representing 31 per cent of the total for the United States—776,304 infants were born alive in 1915, representing a birth rate of 24.9 per 1,000 of population For every state in the registration area and for most of the citites there was a substantial excess of births over deaths, but this excess was most pronounced in those localities in which the proportion of foreign population is largest. The mortality rate of infants under 1 year of age averagedd 100 per 1,000 births, ranging from 70 in Minnesota to 120 in Rhode Island, and, among places having 25,000 inhabitants or more, from 54 in Brookline and Malden, Mass., to 196 in Shenandoah, Pa. These are among the significant facts presented in a preliminary statement just made public by Director Sam L. Rogers of the Bureau of the Census, Department of Commerce.

This statement—giving, as it does, the first Federal statistics of births ever published—makes possible a comparison of birth rates in the registration area of the United States and in foreign countries, and throws light on such matters as the extent to which the populations of the states and cities in the birth-registration area are increasing through excess of births over deaths, the relation between the birth rate and the rate of infant mortality, the relation between the birth rate and the racial composition of the population, etc.

### Comparison of Birth and Death Rates.

The birth rate for the birth-registration area as a whole in 1915—24.9 per 1,000 population—exceeded the death rate for the same area—14 per 1,000—by 10.9 per 1,000, or nearly 78 per cent. That is to say, if the birth and death rates prevailing in that year were to remain unchanged, and if no migration were to take place to or from the area to which the figures relate, its population would increase annually by 10.9 per 1,000, or by nearly 1.1 per cent. The birth rates of the registration states ranged from 21.1 in Maine to 26.7 in Connecticut and Michigan; and the death rates ranged from 10.1 in Minnesota to 16·1 in New Hampshire. The highest death rate was thus much lower than the lowest birth rate. The greatest excess of births over deaths—14.4 per 1,000 population—appears for Minnesota, and the smallest—5..5 per 1,000—for Maine.

The statistics cover 96 cities and towns having, at the last census, 25,000 inhabitants or more. Of these, there were only three—Kingston and Troy, N. Y., and Norristown, Pa.—in which the deaths exceeded the births in 1915, and in each case the excess was small, being greatest—1.1 per 1,000 population—for Troy.

The cities showing the highest five birth rates are: Detroit, Mich., 37.9; Chicopee, Mass., 37.6; Niagara Falls, N. Y., 37·5; New Britain, Conn., 36.4; and Chelsea, Mass., 34.5. The death rates for these cities—15.7, 14.7, 16, 11, and 14.3, respectively—were, with the single exception of that for New Britain, higher than the average for the birth-registration area but were far below the maximum death rate shown for any city in the area—21.7, for Norristown, Pa.

The lowest five birth rates appear for Brookline, Mass., 12.7; York, Pa., 17.5; Kalamazoo, Mich., 18.2; Kingston, N. Y., 18.5; and Troy, N. Y., 18.6. The death rates for the first-named two places were lower than the average for the registration area, and those for the first-named three were lower than the corresponding birth rates; but for Kingston and Troy the death rates—18.6 and 19.7, respectively—exceeded the birth rates.

### Birth Rates of White and Colored Races.

The relation between the birth rate and the constitution of the population in respect of race and nativity is of great interest. For the six cities in the registration area in which the colored population at the last census either numbered more than 10,000 or represented more than 10 per cent. of the total, separate figures are given for the white and colored races; and in all but one of these cities—Washington, D. C.—the birth rates shown for the colored population were lower than those for the whites. It is probable, however, that the registration of births is less nearly complete among colored than among white persons, and that therefore the rates shown for the former class are too low. The death rates for the colored population are higher, and in many cases much higher than those for the whites.

### Births Among Native and Foreign Elements of the Population.

The birth statistics do not, of course, show the number of children per family; but some indication of the fecundity of the native and foreign elements of the population may be obtained from a comparison between the proportion which the number of foreigs parents formed of the total number of white parents to whom children were born in 1915 and the proportion which foreign-born persons represented of the total white population in 1910. From such a comparison it appears that far more births occur annually to foreign-born parents, proportionally to their number, than to native parents. In Connecticut approximately 30 per cent. of the white population in 1910 was of foreign birth, but nearly 63 per cent. of the white parents to whom children were born in 1915 were reported as natives of foreign countries. The corresponding percentages for the other states and the District of Columbia are as follows: Maine, 15 and 28; Massachusette, 31 and 58; Michigan, 21 and 35; Minnesota, 26 and 33; New Hampshire, 22 and 44; New York, 30 and 56; Pennsylvania, 19 and 40; Rhode Island, 33 and 59; Vermont, 14 and 25; District of Columbia, 10 and 17.

Thus, on the assumption that the proportions of native and foreign-born persons in the total white population did not change materially between 1910 and 1915, it appears that the birth rates for the foreign-born population in most of these states and in the District of Columbia are nearly or quite twice as high as the rates for the native and foreign elements combined, and that, on the other hand, the rates for the natives are considerably lower than those for the entire white population, being little more than half as high in the case of Connecticut and less than two-thirds as high in the case of Massachusetts. On the basis of these figures—which of course represent only an approximation to the facts—the excess of the birth rate among the foreign-born population over that among the natives ranges from about 40 per cent. in Minnesota to about 300 per cent. in Connecticut. It should be borne in mind, however, that the proportion of the population in the reproductive period of life is considerably greater for the foreign born than for the natives.

**Infant Mortality.**

The rate of infant mortality—that is, the number of deaths of infants under 1 year of age per 1,000 born alive—is of paritcuarl interest. This rate, for the registration area as a whole, was 100 in 1915. This is practically equivalent to saying that of every ten infants born alive one died before reaching the age of 1 year.

Among the ten states these rates ranged from 70 for Minnesota to 120 for Rhode Island; and among the 96 cities and towns it varied from 54 for Brookline and Malden, Mass., to

196 for Shenandoah, Pa. The maximum rate was thus nearly four times as high as the minimum.

It might be expected that a high rate of infant mortality would accompany a high birth rate, but an examination of the figures fails to disclose any well-defined relationship of this character. Among the states, both the highest and the lowest infant-mortality rates—120 for Rhode Island and 70 for Minnesota—are found in connection with birth rates—23.1 and 24.5 per 1,000 population, respectively—which are below the average for the registration area; and, moreover, the birth rate in the state with the lowest infant mortality is higher than that in the state with the highest infant mortality.

Among the cities and towns the lowest infant-mortality rate—54 per 1,000 births—is shown for both Brookline and Malden, Mass. The former place had the lowest birth rate—12·7 per 1,000 population—given for any city or town in the registration area, but the birth rate of the latter—23.5 per 1,000—was not far below the average for the area. The highest infant-mortality rate—196 per 1,000 births, for Shenandoah, Pa.—is accompanied by a birth rate—32.7 per 1,000 population—which is far above the average, although considerably below the maximum. Of the ten cities in which the birth rates were highest, three show infant-mortality rates lower than the average and of the ten places in which the birth rates were lowest, five show infant-mortality rates higher than the average.

**Birth and Infant-Mortality Rates in Foreign Countries.**

The table given below presents a comparison of the birth and infant-mortality rates for the registration area of the United States with those for foreign countries. The figures for the registration area of the United States relate to 1915, since that is the only year for which such statistics are available; but those for the foreign countries—taken from the annual report of the Registrar-General of England and Wales, 1914—refer in each case to the last calendar year which terminated prior to the outbreak of the European war and for which data as to both classes of rates are available. The birth rates represent the number of infants born alive per 1,000 of population, and the infant-mortality rates represent the number of deaths of infants under 1 year of age per 1,000 born alive.

| Country. | Birth rate. | Infant-mortality rate. |
|---|---|---|
| United States (registration area only: 1915) | 24.9 | 100 |
| England and Wales (1913) | 24.1 | 108 |
| France (1912) | 19.0 | 78 |
| German Empire (1912) | 28.3 | 147 |
| Austria (1912) | 31.3 | 180 |
| Russia in Europe (excluding Finland and the provinces of the Vistula and of the Caucasus: 1909) | 44.0 | 248 |
| Italy (1913) | 31.7 | 137 |
| Spain (1913) | 30.4 | --- |
| Norway (1913) | 25.3 | 65 |
| Sweden (1912) | 23.8 | 71 |
| Denmark (1913) | 25.6 | 94 |
| Belgium (1912) | 22.6 | 120 |
| Holland (1913) | 28.1 | 91 |
| Switzerland (1913) | 23.1 | 96 |
| Japan (1911) | 34.1 | 157 |
| Australia (1913) | 28.3 | 72 |

---

### STRANGE FARMS, UNUSUAL MEANS OF MONEY MAKING.

Turkey has its mosques; Russia has its cossacks; Germany has its U-boats, and Mexico has its fleas; but the United States has the queerest farms in the world.

Out at Pasadena, Cal., Edwin Cawston operates what is perhaps the largest ostrich farm in the world. Of course, it isn't every one who would care to keep ostriches. But Mr.

Cawston doesn't mind it a bit, for he controls a great part of the ostrich plume supply of the world. If you have ever purchased an ostrich plume of the first grade you may have a faint inkling as to how much money can be made from an ostrich farm, if you know how. Once Pennsylvania got the fever and started an ostrich farm up near Sunbury, but the poor, unoffending birds refused to become acclimated; said they were not snowbirds, or something to that effect. Be that as it may, Cawston's ostrich farm remains today the greatest in the world.

At Victoria, in Mexico, there is a parrot ranch. And some distance beyond Los Angeles, Cal., there is an immense pigeon farm. There one will find nearly 15,000 pigeons; indeed, where everybody knows that there is money in pigeons; indeed, where is the school-boy who hasn't kept a few at one time or another? Also, in Colorado there is a bear farm. And somewhere up in Canada is a man who is making money by rearing wolves; the skins bring handsome prices.

Out at Hot Springs, Ark., H. J. Campbell has an alligator farm, which is but another of the American queerest farms in the world. But down in Florida, where the alligator grows, the farmers used to shoot the whole blooming family. It is said that between 1890 and 1900 more than 3,000,000 saurians were killed. Of course, perhaps there was ample reason for this wholesale butchery. The alligators seemed to take great delight in depleting the farmers' herds of cattle. Even the docile cow was not immune. Naturally, making away with the alligators in wholesale lots caused a shortage in alligator skin, and the leather manufacturers felt the pinch. Alligator farms were the result.

And Mr. Campbell goes Dame Nature one better—he hatches 'em out in incubators. After they get beyond the stage where they look like woolly worms with iron-clad backs the alligators are allowed to shoot the chutes, play tag and otherwise make the most of life. But eventually—eventually—the sword of not Damocles but Campbell falls. Later, the pride of the family receives as a graduation gift a lovely alligator-skin grip or suitcase, and he and the baggage-smashers, all unmindful of the shattered romance and the pitiful tragedy back of the advent of the grip or suitcase, treat it shamefully. That's life for you.

In Texas the farmer is breeding buffaloes and crossing them with cattle. In Oregon they are raising Chinese pheasants, but the story of how the ostrich was first introduced to America is one that must be told.

In 1882 an unknown soldier of fortune filled the hold of a steamer bound for New York with more than 100 ostriches. Now, these gigantic birds weigh as much as 200 and 300, pounds, even more. They are accustomed to sunlight, the open range and, above all, fresh air. But here they were, packed in illy-ventilated pens in the smelly hold of a tramp steamer. The pitching and tossing of the steamer also was responsible for the death of many of the birds. At any rate but a mere handful of the original shipment arrived in New York. Later they were shipped to San Francisco, and still later to Anaheim, in Lower California.

Terrapin farming is one of the newer industries. Down on the Isle of Hope, Ga., is one of the greatest of all terrapin farms. And the United States bureau of fisheries has been studying the diamond-back terrapin for the last eight years down at Beaufort, N. C. There terrapin have been in the pounds for more than six years, and the young have long ago reached the age where they can take care of themselves.

William Hagan has an immense fur plant down along the shores of the Delaware—he raises muskrats, and makes money at it. During the season of 1914-15 Mr. Hagan realized more than $2000 clear profit on his immense farm which extends over an area of 614 acres. But muskrat farming is a very strenuous business. In the first place the farmer must wait until fall before the real "farming" takes place. It is then that the skins are at their best. The animals are caught—the greater part of them—by means of stake traps; that is, traps attached to chains, which are in turn attached to stakes. The stakes also serve as a guide. Then, too, the trappers take with them a needle-pointer rapier, used to spear any stray rat which may attempt to flee at the first warning of danger to him or his. And those hip-booted trappers can spear a rat with all the deftness of a William Tell shooting an apple.

If you have never seen a muskrat farm, drop down to Mr. Hagan's place during February—you'll be surprised to see how an "underwater" farm is managed, and you'll hardly be able to believe there are so many muskrats in the world. Some days he averages more than 150, and he has come very near to the 200 mark. Yes, there's lots of money in muskrat farming; but unless you've got the constitution of an Alpine chasseur, don't attempt it.

Joseph Matlack, of Moorestown, N. J., owns what is perhaps the largest guinea-pig farm in the world. This much is uncontradictable. He raises more of them than any grower in America, and makes money where others fail. Now, that's something to be proud of. Any man can be a farmer; but to be a successful farmer—well, that's something different. Of course, there are other guinea-pig farms which enrich their owners—lots and lots of them. But in the guinea-pig world Mr. Matlaick is king.—Montreal Pharmaceutical Journal.

---

### MEDICAL CARE OF THE NATIVE ALASKAN.

The problem of caring for the natives of Alaska is among the most difficult matters which confront the government in its relations with the aboriginal tribes.

There is no central point in Alaska, Seattle being the trading center of the territory.

These people are scattered along a water front of more than 5,000 miles. They live in small villages. They are still influenced by the superstitions which have come down to them from the centuries. They hide, rather than seek relief for their ailments, believing that there is some divine retribution in misfortune.

Secretary Lane of the Interior Department, who personally knows every part of Alaska, has given tender consideration to the needs of the native Alaskan, and great improvement has taken place in the care of these people, especially during the past two years.

Syphilis and tuberculosis, here as elsewhere, have wrought sad havoc with the primitive people.

The editor of the Medical Sentinel, in a trip just completed in Alaska, was forcibly impressed by the special interest now being shown by the government in the medical side of care for the natives.

At Juneau, Dr. Douglas Brown, a recent arrival, is in charge of a splendid native hospital just completed by the Interior Department, which looks after fourteen near-by villages. Dr. Brown serves under the Educational Division of the Interior Department, is a civil service employee, and was for some years with Col. Gorgas in the Panama Canal zone.

At Haines a special hospital is soon to be erected for tubercular cases, and soon a colony with every modern equipment will be in operation.

In other portions of Alaska, seven or eight physicians have been put in charge of the medical Indian service, and three other small native hospitals are already maintained by the government in the territory.

An attempt is now being made by Secretary Lane to employ teachers in the educational division, for stations where no doctors are located, who are also trained nurses. These teachers have some special training for emergency medical work, are given a medical and surgical equipment of simple character, and provided with proper instructions for the service along medical lines. As fast as appropriations can be secured, district zones are being organized comprising a neighborhood of native villages, for which a general hospital and a competent physician is supplied.

The insane native has the benefit of care outside of Alaska, where, in a milder climate, the percentage of recoveries is very large. The tubercular insane live in a separate department, at Portland, Oregon, where they enjoy every qualification for modern treatment.

The educational department in these more recent departures seeks, among other things, to educate the natives as to the prevention of tubercular infection. Also as to the dangers of syphilis and its possible cure under appropriate treatment, thereby effecting the lowest possible evil to the living, as well as to the unborn progeny of the native races of Alaska.—Medical Sentinel.

